# Impact of the digital economy on carbon dioxide emissions in resource-based cities

**DOI:** 10.1038/s41598-024-66005-0

**Published:** 2024-07-17

**Authors:** Yiming Kuang, Yaojun Fan, Jie Bin, Min Fan

**Affiliations:** 1 International Business School, Hunan University of Information Technology, Changsha, China; 2 Stamford International University, Petchburi, Thailand; 3 José Rizal University, Manila, Philippines; 4 Lanzhou University, Lanzhou, China

**Keywords:** Digital economy, Resource-based cities, Carbon dioxide emissions, Nonlinear effects, Environmental regulation, Sustainable development, Climate-change mitigation, Sustainability

## Abstract

With the rapid development of the digital economy, its environmental impact, particularly on carbon dioxide emissions in resource-based cities, has emerged as a vital research topic. Resource-based cities, often central to traditional industries, are confronted with the dual challenges of environmental pollution and economic transformation. This study employs empirical analysis to examine the influence of the digital economy on carbon dioxide emissions in these cities. The findings reveal that the digital economy significantly reduces carbon dioxide emissions, with this impact being more pronounced in the early stages of digital economic development and gradually diminishing thereafter. In the mechanism analysis, we found that the digital economy can reduce carbon dioxide emissions in resource-based cities by raising public concern about the environment. Moreover, the study highlights significant variations in carbon reduction effects among different types of resource-based cities, noting that stronger environmental regulations further enhance these effects. These insights not only provide a new theoretical perspective but also offer practical guidance for policymakers in promoting sustainable development within the digital economy.

## Introduction

In the twenty-first century, set against the backdrop of economic globalization and rapid advancements in information technology, the digital economy has emerged as a new driving force of global economic growth. Defined by the International Telecommunication Union (ITU) as economic activities centered around digital information and communication technologies (ICT), encompassing e-commerce and digital transactions^1^, this economic paradigm reshapes traditional business operations and alters the global economic structure and growth patterns^2^. Accompanying the innovation and widespread application of digital technologies, resource-based cities, historically reliant on industries like mining and energy extraction, are now facing unprecedented opportunities for transformation and environmental challenges. According to World Bank data, these cities significantly contribute to global carbon dioxide emissions, exacerbating global warming trends and profoundly impacting local socio-economic development and the quality of life for residents. The rise of the digital economy presents a new pathway to sustainable development, potentially reducing resource consumption and environmental pollution while fostering economic and social progress^3–5^. The topic of this study—The impact of the digital economy on carbon dioxide emissions in resource-based cities—Directly responds to the pressing issues of global climate change and sustainable development.

Carbon dioxide emissions, a central factor in global climate change, have become a critical issue for international communities. Resource-based cities, with their history of heavy industry reliance and resource exploitation, are often associated with high energy consumption and pollution. Their carbon dioxide emissions not only pose a global environmental threat but also impact the sustainable development and health of the cities themselves^6^. Against this backdrop, investigating the impact of the digital economy on carbon dioxide emissions in these cities is particularly pertinent. The digital economy, by integrating information technology with traditional industries, not only promotes industrial optimization and upgrading but also provides new opportunities for urban transformation. For example, the application of digital management and intelligent technologies can improve energy efficiency and reduce unnecessary resource use, thereby lowering carbon dioxide emissions^7^. Additionally, the digital economy stimulates the development of new environmental technologies, such as renewable energy and smart grids, contributing to a low-carbon economic transition^8^. However, the impact of the digital economy is a complex, multifaceted process influenced by technological development, policy environments, market demands, and societal awareness. Moreover, the development of the digital economy itself may introduce new energy demands and environmental challenges, such as the energy consumption of data centers^9^. Therefore, a thorough investigation and understanding of the specific impacts of the digital economy on carbon dioxide emissions in resource-based cities are essential for formulating effective environmental policies and promoting green transformation in these areas.

This study aims to comprehensively explore how the digital economy influences carbon dioxide emissions in resource-based cities. By analyzing the characteristics and developmental trends of the digital economy, as well as its current applications in these cities, the study will reveal the potential impact mechanisms on carbon dioxide emissions and, based on empirical data, analyze its actual effects. The ultimate goal of this research is to propose specific policy recommendations to promote sustainable development in resource-based cities in the digital economy era, effectively reducing carbon dioxide emissions and contributing to global environmental protection.

## Literature review and theoretical analysis

### Literature review

The study of global climate change has centered carbon dioxide emissions as a core issue, with trends and impacts being pivotal in the discourse. Since the industrial revolution, the extensive burning of fossil fuels and changes in land use have led to a significant increase in CO2 emissions, exacerbating the greenhouse effect and triggering a series of environmental issues such as rising global average temperatures, increased frequency of extreme weather events, and rising sea levels^10–12^. The contribution of CO2 emissions to climate change is undeniable. Köne & Büke^11^ employed trend analysis methods to forecast the trends of CO2 emissions related to energy consumption, revealing a close connection between global CO2 emission growth, economic activities, and energy structures. Wei et al.^13^ presented a more precise predictive model for CO2 emissions in Hebei Province, China, using a moth-flame optimization model with random forests and extreme learning machines. Beyond quantitative studies, other research has focused on the global distribution and changing trends of CO2 emissions. For example, Sitch et al.^14^ assessed global and regional trends in land and oceanic carbon exchanges with respect to climate and atmospheric CO2 using dynamic global vegetation models and marine biogeochemistry models, covering the period from 1990 to 2009. Their findings highlighted the significant impact of CO2 absorption interplaying with climate change and variability on the global CO2 cycle. These studies provide a comprehensive understanding of global CO2 emission trends, unveiling the profound impact of industrialization, economic development, and changes in energy structures on global climate change. Understanding these trends is crucial for formulating effective CO2 emission reduction strategies and addressing global climate change.

Resource-based cities, with their high dependency on natural resources, face unique environmental challenges. Their economic models often lead to intense energy consumption and substantial CO2 emissions. As resources gradually deplete, these cities not only experience economic decline and social issues but also environmental degradation, severely hindering the possibility of sustainable development. Athanassiadis et al.^15^ emphasized the significance of cities as complex systems linked to global supply chains and their impact on environmental flows through a territorial and consumption-based assessment of urban environmental performance. This insight offered a new perspective on the environmental challenges of resource-based cities and underscored the importance of global environmental impacts. Zhang et al.^16^ introduced the resource-based cities sustainability index and resource-based city coordination index (RCCI) as comprehensive tools for assessing the environmental, social, and economic dimensions of cities. These indices aid in understanding the developmental trends and challenges of cities from multiple angles. Furthermore, Tan et al.^17^ delved into the transformational performance of resource-based cities in Northeast China, while Hou et al.^18^ analyzed the impact of environmental regulation and financial pressure on industrial ecological efficiency, revealing the potential positive role of appropriate environmental policies in enhancing the ecological efficiency of resource-based cities. Collectively, these studies paint a detailed picture of the environmental challenges and opportunities for transformation faced by resource-based cities, highlighting their complexity in economic, social, and environmental aspects.

The rapid development of the digital economy presents new opportunities for global environmental benefits. Advances in information and communication technology have positioned the digital economy as a representative of environmentally friendly economic models. Compared to traditional economic models, the digital economy features low energy consumption and reduced pollutant emissions. Therefore, exploring the impact of digital economy development on ecological performance is of significant importance. Shen et al.^19^ found that the digital economy significantly enhances the ecological performance of Chinese provinces, exhibiting strong externality in space, that is, creating spillover effects on neighboring regions. Linkov et al.^20^ highlighted the crucial role of digital technologies in enhancing connectivity and networking in communication, services, and trade, potentially impacting the response to sustainability challenges. Creutzig et al.^21^ emphasized the profound impact of the indirect and systemic effects of digitalization in reshaping the relationships among humans, technology, and the planet, by examining the past, present, and future of digitalization. Herman^22^ explored the interrelation between digital entrepreneurship and the sustainable development goals of EU countries, finding that digital entrepreneurship positively influences the achievement of these goals. Yu and Wan^23^ focused on how the digital economy impacts CO2 emissions in Chinese cities, discovering that while the digital economy initially intensifies CO2 emissions, it can aid in achieving urban decarbonization goals once a certain level is reached. These studies demonstrate that the digital economy, while promoting the diversification of the economic structure in resource-based cities, also reduces environmental impacts through intelligent management and technological innovation, offering new possibilities for enhancing urban ecological performance. Additionally, existing research has revealed several main pathways through which the digital economy reduces carbon emissions. These include promoting industrial structure upgrades^24,25^, driving green innovation^26^, enhancing production and consumption efficiency^25,27^, facilitating the agglomeration of producer services^27^, and developing digital finance^25^. The digital economy contributes to emissions reduction by optimizing resource allocation and reducing transaction costs^28^. However, the specific mechanisms in different fields require further clarification and quantification.

In summarizing these studies, we observe the immense potential of the digital economy in promoting sustainable development. However, existing research also has limitations in methodological and theoretical frameworks, such as an inadequate depth of understanding of the complex relationship between the digital economy and environmental sustainability, and a need for comprehensive exploration of the dynamic relationship and potential mechanisms between digital economy development and CO2 emissions. Future research should employ more comprehensive and systematic approaches to better understand the complex relationship between the digital economy and environmental sustainability. This understanding is vital for formulating related policies and strategies, providing a scientific basis for decision-making that supports sustainable development in the context of the digital economy.

### The impact of the digital economy on carbon emissions in resource-based cities

Resource-based cities, characterized by their economic reliance on natural, especially mineral, resources, face significant challenges in transitioning to new economic models. The development of the digital economy offers these cities fresh opportunities for growth. By introducing efficient and intelligent technological solutions, it not only fosters economic diversification but also enhances resource utilization efficiency and reduces carbon emissions^29,30^.

Initially, the digital economy enhances energy efficiency significantly by integrating advanced information technologies such as the Internet of Things, big data analytics, and artificial intelligence. In resource-based cities, these technologies optimize energy distribution and consumption, particularly in heavy industries and energy-intensive sectors^7,31^. For instance, real-time data analysis and intelligent forecasting can reduce electrical waste and boost production efficiency^32^. Smart sensors and automation systems precisely control energy use throughout the production process, thereby curtailing unnecessary consumption and carbon emissions^8^. Additionally, digital technologies play a crucial role in integrating and managing renewable energy sources^33^. Advanced data analytics and smart grid technologies enable more effective integration of renewable sources like solar and wind energy into the power grid, reducing reliance on fossil fuels. This not only improves energy utilization efficiency but also helps balance supply and demand, ensuring the stability of energy supply^34^. Moreover, digital management systems optimize energy storage, enhancing the proportion of renewable energy in the energy mix^9^. Finally, the digital economy provides resource-based cities with the opportunity to shift from traditional heavy industry and resource-dependent economies to more service and technology-oriented economies^35^. This transformation involves new business models and job opportunities in areas such as digital services, e-commerce, remote work, and innovative technology enterprises. These fields typically have lower carbon emission intensities compared to traditional resource extraction industries. Furthermore, the digital economy also fosters innovation and entrepreneurship, creating a fertile ground for the development of low-carbon technologies and sustainable solutions^36^.

Therefore, the digital economy offers a new pathway for transformation and development in resource-based cities. By improving energy efficiency, promoting the use of clean energy, and optimizing the economic structure, the digital economy can help these cities reduce carbon emissions and achieve sustainable development. This paper proposes the following research hypothesis H1:

#### H1

 The digital economy contributes to the reduction of carbon emissions in resource-based cities.

### The digital economy, public environmental awareness, and carbon emissions in resource-based cities

As environmental issues, such as global warming and resource depletion, garner increasing global attention, the public's awareness of environmental protection has significantly risen^37^. Media coverage, activities by environmental organizations, and governmental policy promotions are continually elevating public consciousness towards environmental conservation^38^. This heightened environmental awareness has spurred demand for sustainable development and green technologies, thereby influencing consumer behavior, corporate strategies, and policy making^39^. In resource-based cities, the high level of public concern for environmental protection plays a crucial role in reducing carbon emissions^40^. This concern promotes the spread of environmental education, enhancing societal awareness of climate change issues and inspiring individuals and collectives to adopt more eco-friendly behaviors in daily life. The increasing public demand for clean energy and green technologies drives businesses and governments to invest in low-carbon solutions, such as renewable energy and energy-saving technologies, accelerating the green transformation process of cities^41^. Moreover, public participation has facilitated the development and implementation of environmental policies, creating a favorable policy environment for the development of a low-carbon economy^42^. These factors, working together, not only drive the reduction of carbon emissions in resource-based cities but also lay the foundation for achieving sustainable development goals.

Furthermore, the digital economy, through its extensive information dissemination and efficient technological applications, has had a positive impact on the high level of public concern for environmental protection^43^. Firstly, digital platforms offer a vast space for the rapid spread of environmental information and knowledge, enhancing public understanding and awareness of environmental issues^44^. Additionally, digital technologies, such as big data analysis and artificial intelligence, provide precise data support for environmental monitoring, allowing the public to gain a more intuitive understanding of environmental changes and pollution issues^45^. Social media and online forums, among other digital platforms, have stimulated public participation in environmental discussions and activities, promoting the enhancement of environmental consciousness and the initiation of actions^46^. This increase in public environmental concern driven by the digital economy not only deepens societal recognition of the importance of environmental protection but also establishes a solid social foundation for achieving more sustainable development goals.

Therefore, enhancing public environmental awareness is a critical pathway through which the digital economy reduces carbon emissions in resource-based cities. This paper proposes the following research hypothesis H2.

#### H2

 The digital economy reduces carbon emissions in resource-based cities by enhancing public environmental awareness.

### Digital economy, non-linear effects, and carbon emissions in resource-based cities

The impact of the digital economy on carbon emissions in resource-based cities is not constant but exhibits clear non-linear characteristics. This non-linear effect manifests as a more pronounced reduction in carbon emissions during the early stages of digital economy development; however, as the digital economy matures, this emission reduction effect gradually diminishes^6,7^.

In the early stages of the digital economy, resource-based cities achieve significant improvements in production efficiency and optimization of energy use by adopting innovative digital technologies like cloud computing, big data, the Internet of Things, and artificial intelligence. The application of these technologies leads to substantial gains in energy efficiency, particularly in industrial production where the introduction of intelligent systems and automation significantly reduces energy wastage and optimizes production processes. This reduction in raw material consumption lowers energy demands and associated carbon emissions^29,31^. Additionally, the replacement of outdated and high-energy-consuming equipment and processes further strengthens the impact of energy conservation and emission reduction^30^. During this transformative period, policy support from governments and private sectors, such as tax incentives, financial subsidies, and technology development funding, play a pivotal role. These policies not only accelerate the dissemination and adoption of digital technologies but also bolster the transition of resource-based cities towards more efficient and eco-friendly directions^32^. Thus, the initial stages of the digital economy significantly impact the reduction of carbon emissions in resource-based cities, a result of both technological advancements and the combined effects of policy and socio-economic factors.

In the mid to later stages of digital economy development, the diminishing effect on carbon emission reduction in resource-based cities reflects a complex interplay of economic and technological factors. During this phase, although technological advancements continue, the marginal benefits of improving energy efficiency and reducing emissions decrease due to the widespread adoption of initially easier innovations, making further technological improvements more challenging^36^. Moreover, with the rise of emerging digital economy sectors like data centers and cloud services, the overall energy demand may increase, especially in cities where the energy structure is still predominantly fossil fuel-based^33^. Concurrently, the rapid growth of electronic waste presents new challenges for environmental management^47^. Hence, in the mid to later stages of the digital economy, resource-based cities face the necessity of recalibrating strategies to focus on more efficient energy utilization technologies, promoting the development and application of clean energy, and effectively managing electronic waste, ensuring environmental sustainability while pursuing economic growth^8^. The challenges of this phase call for a more comprehensive and forward-looking strategic plan to adapt to the new stage of technological development and its changing environmental impacts.

Therefore, the effect of the digital economy on reducing carbon emissions in resource-based cities changes over time. To fully leverage the potential of the digital economy in reducing carbon emissions, a deep understanding of this non-linear effect is essential, and policies and measures must be adjusted and formulated according to different stages of development. This paper proposes the following research hypothesis H2:

#### H3

 The effectiveness of the digital economy in reducing carbon dioxide emissions in resource-based cities diminishes over time.

### Methodology

#### Variable selection

Carbon dioxide emissions (CO2). Drawing from the research of Cong et al.^48^, this study measures the CO2 emissions in resource-based cities. Specifically, the study assesses CO2 emissions from three scopes: Scope 1, Scope 2, and Scope 3, with the total emissions being the sum of emissions from all three scopes. Scope 1 refers to all direct emissions within the city's jurisdiction, including energy activities (industrial, transportation, and building), industrial processes, agriculture, land-use changes and forestry, and waste management activities. Scope 2 encompasses indirect emissions related to energy occurring outside the city's jurisdiction, primarily emissions from purchased electricity, heating, and/or cooling for city consumption. Scope 3 includes other indirect emissions caused by internal city activities but occurring outside the jurisdiction and not covered by Scope 2, such as emissions from the production, transportation, use, and waste management of goods purchased from outside the city's area. The precision of this method lies in its specific consideration of different sources of emissions. Direct emissions data from Scope 1 reflect emissions from internal city activities, while indirect emissions data from Scopes 2 and 3 reveal the city's impact on the external environment. This segmentation provides deeper insights into urban carbon emissions. For consistency in units, the total emissions are logarithmically transformed.

Digital economy (de). Following the methodology of Zhao et al.^49^, this study measures the level of the digital economy in resource-based cities using principal component analysis. Specifically, the study measures the comprehensive development level of the digital economy from two aspects: internet development and the inclusiveness of digital finance. This multidimensional measurement more accurately reflects the complexity and diversity of the digital economy, ensuring the depth and breadth of the research. For the measurement of internet development at the city level, indicators such as internet penetration rate, relevant employment status, related output, and mobile phone penetration rate are used. For digital finance development, the China Digital Inclusive Finance Index is used, which is jointly compiled by the Digital Finance Research Center of Peking University and Ant Group. Through principal component analysis, the data from these five indicators are standardized and dimensionally reduced, resulting in a comprehensive development index for the digital economy, denoted as de.

The study uses economic development level (EDL), population density (PD), science and technology expenditure (STE), foreign direct investment (FDI), industrial structure level (ISL), and financial development level (fin) as control variables (see Table 1). These variables allow for a more comprehensive consideration of other factors affecting air pollution, thereby more accurately identifying the relationship between digital inclusive finance and air pollution.

**Table 1:** Variable definition.

	Variable name	Variable symbol	Measure of variable
Dependent variable	Carbon dioxide emissions	CO2	Carbon dioxide emissions
Independent variable	Digital economy	DE	It is calculated by principal component analysis
Control variables	Economic development level	EDL	Per capita GDP logarithm
	Population density	PD	The logarithmic value of population per square kilometer
	Science and technology expenditure	STE	The proportion of technology expenditure to fiscal expenditure
	Foreign direct investment	FDI	The proportion of foreign direct investment in GPD
	Industrial structure level	ISL	The proportion of output value of the tertiary industry to GDP
	Financial development level	FDL	The proportion of loan balance from financial institutions to GDP

#### Model design

As in previous studies, considering that individual and time factors may affect the regression results, this paper uses the research data of 107 resource-based cities in China to construct the following two-way fixed-effect model to test the specific impact of digital economy on carbon dioxide emissions of resource-based cities. There are significant differences in the geographical location, economic development level, industrial structure and policy environment of resource-based cities. These unobserved heterogeneity may have an impact on CO2 emissions in cities. By introducing city fixed effect and time fixed effect, bidirectional fixed effect model can effectively control these potential unobserved factors and reduce estimation bias.

1 $${co2}_{i,t}={\alpha }_{0}+{\alpha }_{1}{de}_{i,t}+\delta X+{\gamma }_{i}+{\omega }_{t}+{\varepsilon }_{i,t}$$

Among them, CO2 represents the level of air pollution at the city level, de represents the level of digital financial inclusion in the city, and X represents the control variables, which are respectively economic development level (EDL), population density (PD), science and technology expenditure (STE), foreign direct investment (FDI), industrial structure level (ISL), and financial development level (FDL). In addition, ${\gamma }_{i}$ and ${\gamma }_{i}$ represent the fixed effect of urban individuals and the fixed effect of time respectively.

To investigate the mechanism through which the digital economy impacts carbon emissions in resource-based cities, and given the significant causal flaws of traditional mediation models^50^, this study adopts the improved model approach proposed by Jiang^50^ and Hu^51^ for examining the mechanism of public environmental awareness. Herein, "car" represents public environmental awareness, with Eq. (2) being identical to Eq. (1).

2 $${co2}_{i,t}={\alpha }_{0}+{\alpha }_{1}{de}_{i,t}+\delta X+{\gamma }_{i}+{\omega }_{t}+{\varepsilon }_{i,t},$$

3 $${\text{car}}_{i,t}={\alpha }_{0}+{\alpha }_{1}{de}_{i,t}+\delta X+{\gamma }_{i}+{\omega }_{t}+{\varepsilon }_{i,t},$$

4 $${co2}_{i,t}={\alpha }_{0}+{\alpha }_{1}{\text{car}}_{i,t}+\delta X+{\gamma }_{i}+{\omega }_{t}+{\varepsilon }_{i,t},$$

In order to deeply analyze the nonlinear characteristics of the impact of digital economy on carbon dioxide emissions in resource-based cities, the panel threshold model is adopted for empirical test. This model choice is based on its advantages in dealing with nonlinear relationships in complex economic data, and is particularly suitable for exploring and verifying nonlinear relationships between variables. After threshold test, the single threshold model is selected. The specific model is as follows.

5 $${co2}_{i,t}={\alpha }_{0}+{\delta }_{1}{de}_{it}\times I\left(de\le {\gamma }_{1}\right)+{\delta }_{2}{de}_{it}*I\left(de>{\gamma }_{1}\right)+\delta X+{\gamma }_{i}+{\omega }_{t}+{\varepsilon }_{i,t},$$

where I (·) is the indicative function and ${\upgamma }_{1}$ is the threshold value, that is, when de is less than ${\upgamma }_{1}$, the impact coefficient of digital economy on carbon dioxide emissions of resource-based cities is ${\updelta }_{1}$; when de is greater, the impact coefficient of digital economy on carbon dioxide emissions of resource-based cities is ${\updelta }_{2}$.

## Results

### Data description

Given the availability and continuity of city data, this study selects data from 107 resource-based cities in China, as listed in the "National Plan for Sustainable Development of Resource-Based Cities (2013–2020)". The CO2 data for these cities are sourced from various Chinese statistical yearbooks and related statistical materials, including the "China Energy Statistical Yearbook", "China Industrial Statistical Yearbook", "China Agricultural Statistical Yearbook", "China Animal Husbandry Yearbook", and "China Forestry and Grassland Statistical Yearbook". The data on the digital economy are derived from the "China City Statistical Yearbook" and the "China Digital Inclusive Finance Index (2011–2021)", compiled jointly by the Digital Finance Research Center of Peking University and Ant Group. Other city data are also sourced from the "China City Statistical Yearbook". Additionally, missing values are imputed using linear interpolation. The descriptive statistics, as summarized in Table 2, provide an overview of the variables across 1177 observations. The mean CO2 emissions stand at 17.193 with a standard deviation of 0.436, indicating a relatively concentrated distribution around the mean. The digital economy (DE) shows a mean value of 0.511 with a more substantial standard deviation of 0.258, reflecting a wider variation in the development level of the digital economy across different cities. The economic development level (EDL) has a mean of 10.637, showcasing the general economic status of the cities in the dataset. Population density (PD), technological expenditure (STE), foreign direct investment (FDI), industrial structure (ISL), and financial development level (FDL) display varied ranges, indicating diverse urban characteristics among the sampled cities.

**Table 2:** Descriptive statistics.

VarName	Obs	Mean	SD	Median	Min	Max
CO2	1177	17.193	0.436	17.361	15.735	17.711
DE	1177	0.511	0.258	0.428	0.114	2.145
EDL	1177	10.637	0.530	10.629	8.773	12.456
PD	1177	5.388	0.936	5.429	2.303	6.999
STE	1177	0.012	0.012	0.008	0.001	0.207
FDI	1177	0.002	0.002	0.001	0.000	0.018
ISL	1177	0.402	0.084	0.400	0.144	0.618
FDL	1177	0.893	0.510	0.817	0.132	9.623

**Figure 1:**
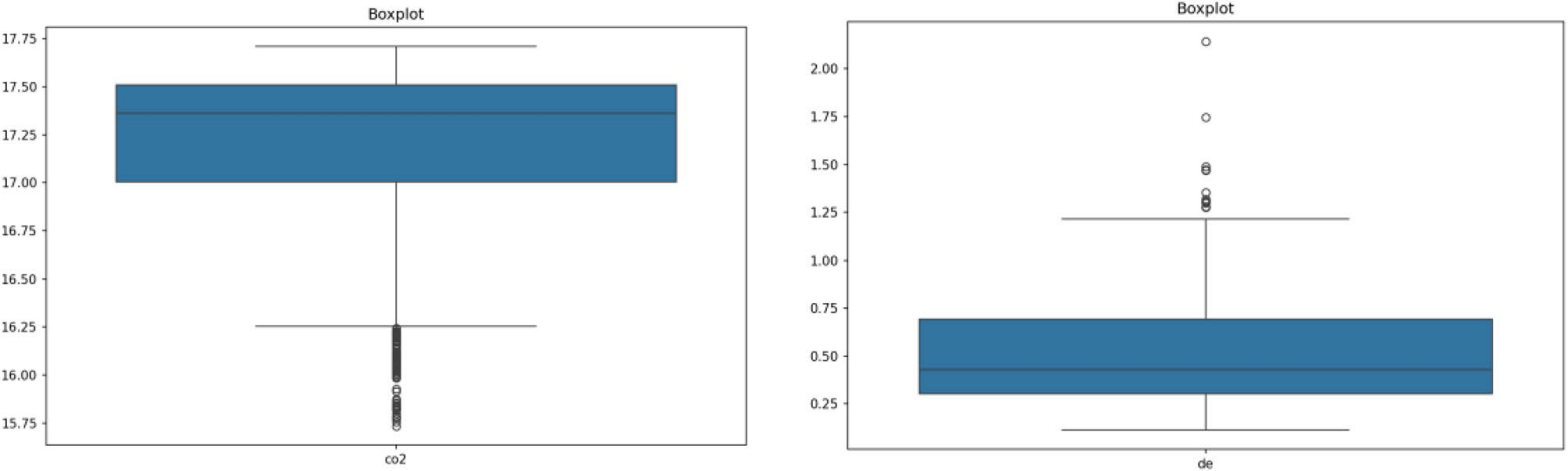
Box plots.

Figures 1 further illustrate the distribution of key variables CO2 and DE through box plots. The left part of Fig. 1 presents the box plot of the CO2 variable. The median CO2 emission is approximately 17.25, indicating that half of the data points are above this value and half are below. The interquartile range (IQR), represented by the box, spans from about 17.00 to 17.50, illustrating the middle 50% of the data. The whiskers extend to approximately 16.00 at the lower end and 17.75 at the upper end. Notably, several data points below the lower whisker signify outliers, showing cities with significantly lower CO2 emissions compared to the majority. The right part of Fig. 1 depicts the box plot of the DE variable. The median value is around 0.50, indicating that half of the observations fall above and half below this value. The IQR stretches from about 0.25 to 0.75. The whiskers extend from approximately 0.00 to 1.00, with several outliers above the upper whisker, indicating cities with notably higher levels of digital economy development. These visualizations enhance the understanding of the dataset's characteristics, offering a more comprehensive view of the relationships and distributions of key variables.

### Baseline regression results

Table 3 presents the test results of the impact of the digital economy on CO2 emissions in resource-based cities. Column (1) shows the regression results without control variables, column (2) includes fixed effects, column (3) adds control variables but not fixed effects, and column (4) includes both control variables and fixed effects. It is found that the coefficient of DE (digital economy) is consistently negative across all models, indicating that the development of the digital economy contributes to the reduction of CO2 emissions in resource-based cities, thereby validating the research hypothesis H1. Specifically, this impact is manifested in several ways: firstly, the digital economy enhances energy efficiency and optimizes energy structure, reducing dependence on traditional high-pollution energy sources and directly decreasing CO2 emissions. Secondly, the development of the digital economy promotes the innovation and application of green technologies, which help reduce carbon emissions in industrial production, transportation, and construction. Additionally, the digital economy changes consumption patterns and production methods, such as e-commerce and remote work reducing the need for physical transportation and associated carbon emissions. Finally, the application of digital technology in environmental monitoring and management improves the precision and effectiveness of policy-making, making the achievement of environmental protection goals more efficient. These findings not only enrich the theoretical understanding of the relationship between the digital economy and environmental protection but also provide empirical evidence for policymakers, indicating specific pathways to achieve environmental sustainability through digital transformation. In this way, the digital economy plays a crucial role in promoting the sustainable development of resource-based cities.

**Table 3:** Baseline regression results.

	(1)	(2)	(3)	(4)
	CO2	CO2	CO2	CO2
DE	− 0.254***	− 0.050*	− 0.135**	− 0.057**
	(0.049)	(0.026)	(0.054)	(0.027)
EDL			0.030	− 0.016
			(0.024)	(0.020)
PD			0.125***	0.373***
			(0.014)	(0.122)
STE			0.634	0.232
			(1.124)	(0.328)
FDI			− 25.595***	1.164
			(5.557)	(2.563)
ISL			0.180	− 0.096
			(0.169)	(0.089)
FDL			0.077***	0.014**
			(0.026)	(0.007)
Control	No	No	YES	YES
City_FE	No	YES	No	YES
Year_FE	No	YES	No	YES
Obs	1177	1177	1177	1177
R2	0.023	0.959	0.096	0.959

The symbols *, **, *** indicate significance at the 10%, 5%, and 1% levels, respectively.

### Robustness test results

#### Replacing the dependent variable

**Figure 2:**
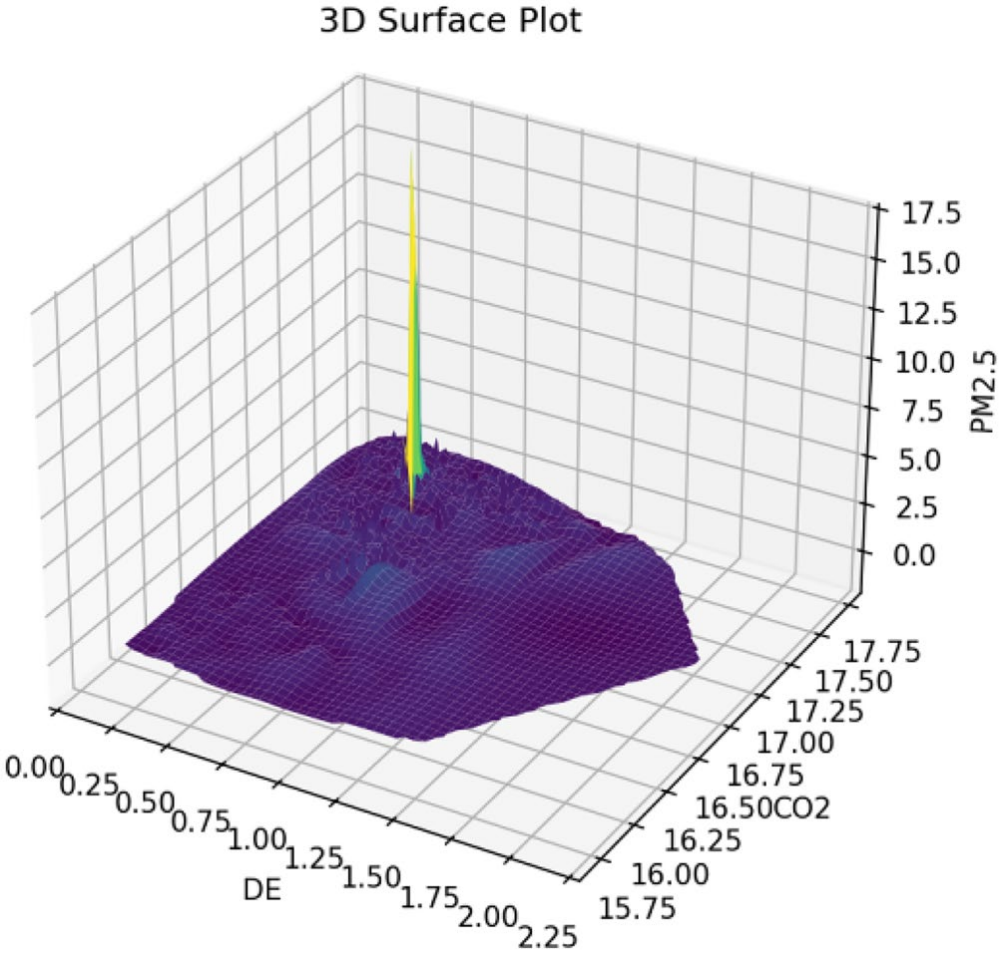
3D surface plot.

Since CO2 and PM2.5 (particulate matter) often originate from the same activities, particularly the burning of fossil fuels (such as coal, oil, and natural gas), and the policies and strategies for reducing these pollutants are often interconnected, PM2.5 is used as a supplementary indicator for CO2 emissions in environmental research of resource-based cities. Using PM2.5 concentration data from the Atmospheric Composition Analysis Group for resource-based cities in China as the dependent variable, the regression results (shown in column 1 of Table 4) indicate a significant negative coefficient for DE, suggesting that the development of the digital economy significantly reduces PM2.5 concentration, indirectly indicating its ability to reduce CO2 emissions. To further validate the robustness of the model results, we employ a 3D surface plot to visualize the relationship between digital economy (DE), CO2 emissions, and PM2.5 concentration. As shown in Fig. 2, the 3D surface plot presents the interaction among these three variables. The X-axis represents the digital economy (DE), the Y-axis represents CO2 emissions, and the Z-axis represents PM2.5 concentration (poll). The 3D surface plot reveals several important observations: the plot indicates that as the digital economy develops, PM2.5 concentration decreases, suggesting that advancements in the digital economy lead to cleaner technologies and more efficient resource utilization, thereby reducing air pollution. Additionally, the plot shows that higher CO2 emissions are associated with higher PM2.5 concentrations, consistent with the fact that both pollutants often result from similar sources, such as fossil fuel combustion.

**Table 4:** Robustness test results.

	(1)	(2)	(3)	(4)
	PM2.5	CO2	DE	CO2
DE	− 0.044***			− 0.079**
	(0.012)			(0.039)
DE2		− 5.931**		
		(2.721)		
iv			0.304***	
			(0.007)	
Control	YES	YES	YES	YES
City_FE	YES	YES	YES	YES
Year_FE	YES	YES	YES	YES
Obs	1177	1177	1050	1050
R2	0.932	0.959		0.959

The symbols **, *** indicate significance at the 5%, and 1% levels, respectively.

#### Replacing the core explanatory variable

In the baseline regression, the study uses principal component analysis to measure the level of digital economy development. The study then recalculates the level of the digital economy in resource-based cities using the entropy method, an objective weighting method that determines the weights of various indicators based on the dispersion of the data. This method reduces the impact of subjective judgment on weight distribution, enhancing the objectivity and scientific nature of the measurement. The regression results using the entropy method (shown in column 2 of Table 4) still show a significant negative coefficient for DE2, indicating that the development of the digital economy continues to reduce CO2 emissions in resource-based cities, confirming the robustness of the study's conclusions.

#### Instrumental variable method

To address potential endogeneity problems in the model, such as reverse causality, the study employs the instrumental variable method. The key to choosing an instrumental variable is that it must be correlated with the explanatory variable in the model while being uncorrelated with the model's error term. Following the research approach of Li et al.^52^ and Lewbel^53^, the study constructs an instrumental variable for the digital economy using the comprehensive share movement instrumental variable method. Specifically, the annual growth rate of the average digital economy level of resource-based cities is calculated as the overall growth rate (shift). Then, the average digital economy level of other resource-based cities within the same province as each city in the previous year is calculated, representing the initial share composition (share). The product of shift and share represents the simulated incremental value of the digital economy for each resource-based cities in each year. Finally, the cubic deviation of each city's digital economy level from this simulated increment (iv) is calculated and used as the instrumental variable for the digital economy in resource-based cities. This instrumental variable, being independent of external factors, satisfies the relevance condition for instrumental variables, and its construction using the movement of shares and previous year's samples effectively enhances its excludability.

The two-stage least squares regression results are shown in columns (3) and (4) of Table 4. Column (3) presents the first-stage regression results, where the coefficient of iv is significantly positive, confirming the relevance of the instrumental variable. Additionally, the F-statistic value of 1927.24 passes the weak instrumental variable test. Column (4) shows the second-stage regression results, where the coefficient of DE remains significantly negative. This outcome reaffirms the robustness of the study’s conclusions.

### Mechanism examination results

To test whether the digital economy can reduce carbon emissions in resource-based cities by enhancing public environmental awareness, a regression analysis was conducted on the mechanism examination model. Drawing from the study by Wu et al.^54^, this paper utilizes the Baidu environmental pollution search index to depict public environmental awareness. With the development of the internet, data based on web search behavior has become a novel indicator, capable of reflecting the public's focus and behavioral intentions timely and accurately. As China's largest Chinese language search engine, Baidu offers extensive coverage and high data availability. Analyzing search frequency and geographical location effectively captures and compares the level of environmental concern across different regions in China. Therefore, using the Baidu environmental pollution search index provides a more direct understanding of the public's concern for air quality and environmental pollution. The regression results, as shown in Table 5, indicate that in column (1), the coefficient of "de" (digital economy) is significantly positive, suggesting that the digital economy significantly promotes an increase in public environmental awareness. In column (2), the coefficient of "car" (public environmental awareness) is significantly negative, indicating that public environmental awareness significantly reduces carbon emissions in resource-based cities. Through this mechanism examination, not only is the hypothesis that the digital economy reduces carbon emissions in resource-based cities by increasing public environmental awareness validated, but the positive role of the digital economy in environmental protection is also demonstrated. These results offer valuable insights for policymakers, highlighting that promoting the development of the digital economy can effectively enhance public environmental consciousness, thereby facilitating the green transformation and sustainable development of resource-based cities.

**Table 5:** Mechanism test results.

	(1)	(2)
	car	CO2
DE	1.568**	
	(0.782)	
Car		− 0.002*
		(0.001)
Control	YES	YES
City_FE	YES	YES
Year_FE	YES	YES
Obs	1176	1176
R^2^	0.838	0.959

The symbols *, ** indicate significance at the 10% and 5% levels, respectively.

This finding aligns with existing environmental economics theories, which posit that public participation and awareness are crucial for the success of environmental protection. The development of the digital age provides new channels and opportunities to enhance public environmental awareness, thereby playing a positive role in environmental protection. These outcomes not only strengthen the understanding of the digital economy's impact on environmental mechanisms but also offer important insights for formulating effective environmental policies, especially in promoting public participation and raising environmental awareness.

### Non-linear test results

To examine whether the impact of the digital economy on CO2 emissions in resource-based cities exhibits nonlinear characteristics, a threshold regression model was used with the digital economy as the threshold variable. The test results, as shown in Table 6, indicate that a single threshold is significant, while a double threshold is not, suggesting the appropriateness of a single-threshold model with a threshold value of 1.8387.

**Table 6:** Threshold test results.

Threshold variable	Threshold test	F value	P value	Critical value	
				5%	10%
DE	Single threshold test	27.70***	0.000	11.901	10.274
	Double threshold test	10.65	0.200	19.728	13.715

The symbol *** indicates significance at the 1% level.

The panel double threshold model regression results, presented in Table 7, show that when the value of the digital economy is below 1.8387, its impact on CO2 emissions in resource-based cities (i.e. the coefficient of DE) is − 0.063, indicating a strong negative effect. This could be due to the initial stages of the digital economy enhancing energy efficiency, fostering innovation, and optimizing industrial structures, thereby significantly reducing urban carbon emissions. As the value of the digital economy exceeds 1.8387, the coefficient of DE changes to − 0.028. This reduction in the negative impact might be attributed to counteracting effects emerging in the later stages of digital economy development, such as increased energy consumption, intensification of electronic waste issues, and more complex socio-economic interactions, which could weaken the positive environmental impact of the digital economy. These results validate the research hypothesis H2, demonstrating that the influence of the digital economy on reducing CO2 emissions in resource-based cities weakens as it develops.

**Table 7:** Panel threshold model regression results.

	(1)
	CO2
DE($\text{DE}\le 1.8387$)	− 0.063***
	(0.007)
DE($\text{DE}>1.8387$)	− 0.028***
	(0.005)
Control	YES
City_FE	YES
Year_FE	YES
Obs	1177
R2	0.795

The symbol *** indicates significance at the 1% level.

This finding aligns with existing environmental economics theories, particularly those revealing complex relationships between technological innovation and environmental effects. In the context of the environmental Kuznets curve (EKC) theory, which postulates a nonlinear relationship between economic development and environmental quality, this can be interpreted to mean that in the initial stages, technological innovation and efficiency gains are key drivers for environmental improvement. However, as the economy further develops, other factors such as increased energy demand and heightened management complexity start to diminish these positive effects.

### Heterogeneity analysis results

#### Impact across different types of resource-based cities

Considering the significant heterogeneity among different types of resource-based cities, which may respond differently to the digital economy, the study further analyzed the variations in the impact of the digital economy on CO2 emissions across different city types. This heterogeneity might stem from differences in resource dependency, economic structure, development stages, and environmental policies of various types of cities. Based on the categorization in the "National Plan for Sustainable Development of Resource-Based Cities (2013–2020)", cities are classified into growing, mature, declining, and regenerating types, and the impact of the digital economy on CO2 emissions in these different types is examined. The regression results for these types of cities are shown in columns (1) to (4) of Table 8. The results indicate that in declining and regenerating cities, the negative impact of the digital economy on CO2 emissions is more pronounced. This may be linked to a higher dependency on the digital economy in these cities during their transformation process. As traditional resource industries decline in these cities, governments and businesses might be more proactive in adopting digital technologies to promote economic diversification and green transformation. Such transformations could involve developing emerging digital industries, promoting smart manufacturing and services, thereby effectively reducing energy consumption and carbon emissions. In contrast, growing and mature cities might still rely on traditional resource extraction and processing industries, with limited penetration and impact of the digital economy. The challenges faced by these cities in digital transformation could be more pronounced, including building technological infrastructure, training skilled labor, and lack of policy support.

**Table 8:** Heterogeneity analysis results.

	(1)	(2)	(3)	(4)	(5)	(6)
	CO2	CO2	CO2	CO2	CO2	CO2
DE	0.008	− 0.056	− 0.085*	− 0.287**	− 0.073**	− 0.044
	(0.081)	(0.042)	(0.046)	(0.119)	(0.036)	(0.048)
Control	YES	YES	YES	YES	YES	YES
City_FE	YES	YES	YES	YES	YES	YES
Year_FE	YES	YES	YES	YES	YES	YES
Obs	154	583	242	154	654	513
R^2^	0.963	0.955	0.966	0.968	0.963	0.963

The symbols *, ** indicate significance at the 10% and 5% levels, respectively.

These results are consistent with existing urban economics and environmental policy theories, especially in understanding the economic transformation and environmental improvement strategies of resource-dependent cities. They highlight the heterogeneity in how different types of resource-based cities respond to the challenges and opportunities of the digital economy and reveal the potential role of the digital economy in promoting sustainable urban development. Particularly for declining and regenerating cities, the development of the digital economy is not only a crucial pathway for economic transformation but also a key strategy for achieving environmental sustainability.

#### Impact of environmental regulatory intensity

In examining the relationship between the digital economy and carbon emissions in resource-based cities, the rigor of environmental policy implementation emerges as an indispensable factor. The strength and effectiveness of environmental regulations in different regions may significantly influence the outcomes of such studies. As a pivotal policy tool, environmental regulatory intensity plays a crucial role in fostering environmental protection and pollution control^55^. This paper adopts the method of Chen and Chen^56^, utilizing Python to segment and analyze keywords related to environmental regulation (such as environmental protection, pollution, energy consumption, emission reduction, discharge, ecology, green, low-carbon, air, chemical oxygen demand, sulfur dioxide, carbon dioxide, PM10, and PM2.5) in city government reports. The frequency of these keywords, relative to the total word count, is used to categorize cities into groups with high and low environmental regulatory intensity. A city is considered to have high environmental regulatory intensity if it meets or exceeds the median annual level of environmental regulation among cities in its province. The regression results of the high and low environmental regulatory intensity samples are shown in Table 8, columns (5) and (6). The findings reveal that in cities with stronger environmental regulations, the negative impact of the digital economy on carbon emissions is more pronounced. This may be linked to the government's emphasis on environmental protection, efficient resource allocation, and effective pollution control measures in these cities. Here, the digital economy, through its promotion of energy efficiency and clean technology applications, likely receives more policy support and social recognition, thus more effectively reducing carbon dioxide emissions. Furthermore, these results align with the theory of environmental policy and digital economy interaction, underscoring the importance of policy environments in fostering technological innovation and application and achieving environmental goals. In cities with robust environmental regulations, the ecological potential of the digital economy is better realized, as these cities often offer more policy incentives, financial support, and market opportunities for promoting energy-saving and emission-reduction technologies and practices.

This discovery holds significant implications for formulating effective environmental policies and promoting the sustainable development of the digital economy. It suggests that strengthening environmental regulations and integrating them with digital economy development strategies can more effectively reduce urban carbon emissions. Hence, policymakers should consider how to motivate businesses and the public to adopt green technologies and practices through more robust environmental regulatory measures, while simultaneously fostering the healthy development of the digital economy.

## Discussions

This paper thoroughly explores the impact of the digital economy on carbon emissions in resource-based cities, yielding numerous meaningful insights. The research found that the development of the digital economy significantly reduces carbon emissions in these cities, thus confirming hypothesis H1. This result aligns with the study by Song et al.^57^, which noted a significant positive impact of the digital economy on ecological performance across various provinces in China. The digital economy, particularly through innovations in cloud computing, big data, and the Internet of Things, has markedly improved energy use efficiency. These technologies have been instrumental in optimizing production processes and reducing energy waste. For example, the application of smart grids and data analysis tools has effectively assisted city managers in more precisely forecasting and adjusting energy needs, thereby reducing unnecessary energy consumption and consequently aiding in lowering carbon dioxide emissions^3,29^. This finding highlights the necessity of continuing to deepen and expand the application of digital technologies in achieving both economic development and environmental protection.

In discussing the results of the mechanism examination, we find that the digital economy significantly promotes the reduction of carbon emissions in resource-based cities by enhancing public environmental awareness. This outcome underscores the dual role of the digital economy in environmental protection: firstly, by broadening public cognition and concern for environmental issues through the extensive application of information technology and online platforms; and secondly, this heightened environmental consciousness further motivates the public to adopt more eco-friendly lifestyles and consumption behaviors, thereby exerting a direct negative impact on urban carbon emissions. Moreover, our research provides vital insights for policymakers, suggesting that fostering the development of the digital economy and leveraging it to enhance public environmental awareness can serve as an effective strategy for achieving sustainable development and reducing carbon emissions in resource-based cities. Therefore, governments and relevant institutions should recognize the potential of the digital economy in raising public environmental consciousness and promoting environmental sustainability. By formulating supportive policies and measures, they can strengthen this positive feedback loop.

Moreover, the study reveals that as the digital economy develops, its impact on carbon dioxide emissions exhibits a distinct non-linear characteristic. In the early stages of digital economy development, its contribution to improving energy efficiency and optimizing resource utilization is significant, leading to a sharp decline in carbon emissions^58,59^. However, as time progresses, this reduction effect gradually weakens, due to several factors. Further exploration into the non-linear relationship between the digital economy and carbon dioxide emissions necessitates considering deeper factors. Initially, there's the evolution and updating of digital technologies; over time, the early introduced digital technologies might reach their peak in energy efficiency improvement^60^. Newer generations of technology, such as more advanced artificial intelligence and IoT solutions, need to be developed and adopted to maintain the momentum of emission reduction^61^. Additionally, the carbon footprint of digital infrastructure itself, including the energy demands of data centers and network equipment, which increase with the expansion of the digital economy, may partially offset the energy-saving effects of digitalization^62,63^. Therefore, promoting the use of clean energy and improving the energy efficiency of data centers becomes crucial. Moreover, the development of the digital economy and changes in industrial and consumption patterns may also impact its emission reduction effects. Digitalization could lead to more efficient production and service models, but it might also generate new energy demands and consumption patterns, such as increased carbon emissions related to logistics and transportation due to the growth of online shopping and digital services^30^. Hence, to maximize the potential of the digital economy in reducing carbon dioxide emissions, a comprehensive strategy is needed. This strategy should include continuous technological innovation and upgrades, optimization of digital infrastructure's energy efficiency, and monitoring and managing the environmental impacts of emerging consumption patterns. Such a comprehensive approach will help resource-based cities more effectively utilize digital transformation to achieve sustainable development goals^64,65^.

In the in-depth discussion on the "differential impacts on different types of cities," this paper focuses on the influence of the digital economy on carbon dioxide emissions in resource-based cities at different stages of development. Initially, growth-oriented cities, usually in rapid industrialization and urbanization phases, might experience significant increases in energy demand. In these cities, the proliferation of the digital economy can effectively optimize energy distribution and consumption patterns, thus aiding in controlling the growth of carbon emissions^29,30^. In contrast, mature cities, with more stable industrial and energy structures, experience the impact of the digital economy predominantly in enhancing the energy efficiency of existing industries and in promoting the application of efficient, low-carbon technologies^23,47^. In these cities, the effects of digital transformation may be more pronounced due to the existing infrastructure and technological level providing a solid foundation for the integration of new technologies. For declining or regenerating cities, the digital economy could become a key driver for transformation and revival. In these cities, the introduction of innovative digital technologies and models can not only help improve economic conditions but also achieve environmental sustainability in the process^3,8^. Overall, different types of cities face various opportunities and challenges in utilizing the digital economy to achieve carbon emission reduction. Therefore, devising digitalization strategies suited to the characteristics of each city type is vital for realizing their respective environmental and economic goals.

In the discussion of how environmental regulation enhances the digital economy's emission reduction effect, it is imperative to consider the interplay between policy and technology. Strengthening environmental regulation can provide more robust support and guidance for the ecological applications of the digital economy. For instance, by establishing stringent emission standards and offering financial incentives, governments can encourage enterprises to adopt low-carbon technologies and improve production processes. Furthermore, environmental regulation can also promote investments in clean energy and energy-efficient technologies, such as smart grids and renewable energy sources^66^, by both public and private sectors^67^. Additionally, stronger environmental regulation can raise public awareness of the importance of sustainable development, fostering a preference among consumers and businesses for eco-friendly digital solutions. When enterprises and consumers recognize that environmentally friendly behaviors not only comply with regulatory requirements but also bring economic benefits, they are more likely to actively engage in the digital economy transformation. Therefore, a synergistic development of effective environmental regulations and the digital economy can not only accelerate the achievement of carbon emission reduction goals but also propel the economy and society towards a more sustainable trajectory^61,68^.

This paper offers important insights into the relationship between the digital economy and environmental improvement, and energy transformation in resource-based cities. The contributions of the study are manifold. Firstly, it adopts an interdisciplinary approach, integrating theories and empirical methods from economics, environmental science, and information technology, providing a new perspective on the digital economy's environmental impact. Secondly, the research reveals how public environmental awareness is enhanced through the development of the digital economy and further affects the level of urban carbon emissions. This finding highlights the potential role of the digital economy in raising public environmental consciousness and promoting sustainable environmental development. Additionally, the study examines the role of environmental regulation in the relationship between the digital economy and carbon emissions, offering evidence to strengthen the environmental protection effects of the digital economy through enhanced environmental regulations. By conducting a segmented analysis of different types of resource-based cities, the research demonstrates the regional heterogeneity of the digital economy's impact, revealing differences in environmental effects across growing, mature, declining, and regenerating cities. Lastly, the study also investigates the long-term effects and dynamic changes of the digital economy on carbon emissions in resource-based cities, providing significant evidence for understanding the sustained impact of the digital economy. These contributions not only enrich the field of study concerning the digital economy and environmental sustainability but also offer scientific bases and practical guidance for policy formulation.

## Conclusion

This study conducts an in-depth analysis of the impact of the digital economy on carbon dioxide emissions in resource-based cities. Our findings reveal that digital technologies, particularly in the realms of intelligent energy management and efficient logistics, have significantly enhanced energy efficiency, thereby reducing carbon emissions in these cities. In addition, we found that the digital economy can reduce CO2 emissions in resource-based cities by raising public awareness of the environment. We also observed a non-linear characteristic in the relationship between the digital economy and carbon emissions, indicating that while initial emission reduction effects are substantial, these effects tend to weaken as technologies mature and markets become saturated. Furthermore, different types of resource-based cities exhibit significant variations in their digital economic transformation. Growth-oriented cities, due to their rapid development needs, are quicker in adopting and applying innovative technologies, whereas mature cities have leveraged their existing infrastructure for more efficient energy utilization. The strengthening of environmental regulation has also proven crucial in promoting the ecological benefits of the digital economy, encouraging technological innovation and market adaptation. The findings of this study not only provide new insights for the theoretical field but also offer practical guidance for policymakers in formulating environmental policies and promoting the sustainable development of the digital economy. Future research could expand the data scope, delve deeper into the effectiveness of digital technology applications in different economic sectors and lifestyles, and examine their impact on other environmental indicators.

### Policy recommendations

Based on the main findings of this study, the following policy recommendations are proposed:

Firstly, the government should further leverage the key role of the digital economy in promoting the sustainable development of resource-based cities. This includes significantly increasing investment in digital infrastructure construction such as smart grids and industrial internet of things (IoT), encouraging enterprises to adopt digital technologies for energy conservation and emission reduction, supporting the development of innovative digital technology companies, and using digital technology to improve urban environmental monitoring and precise governance capabilities.

Secondly, the government should enhance public awareness of environmental protection through Internet propaganda, public education activities, and incentive mechanisms, to fully utilize the potential of the digital economy in raising environmental consciousness. This would not only motivate public participation in energy saving and emission reduction but also strengthen societal oversight of corporate and governmental environmental responsibilities.

Additionally, for resource-based cities at different stages of development, the government should formulate differentiated strategies for the development of the digital economy. For rapidly developing cities, priority should be given to promoting digitalization in areas such as intelligent manufacturing; for mature cities, the integration of digital technology with traditional industries should be strengthened; and for transforming cities, the development of the digital economy could become a key breakthrough for economic diversification and green transition.

Lastly, the government should strengthen environmental regulation by enacting stricter emissions standards, environmental tax policies, and increasing support for green technology research and development, to create a conducive environment for the development of the digital economy in resource-based cities. At the same time, communication and collaboration between governments, enterprises, and the public should be enhanced to create a social atmosphere conducive to the synergistic development of the digital economy and environmental protection.

Implementing the above policy recommendations will better harness the role of the digital economy in promoting the sustainable development of resource-based cities and make a positive contribution to addressing global climate change.

### Research limitations

Addressing the limitations and future research directions of this study, we first acknowledge that although this research provides an initial understanding of the relationship between the digital economy and carbon dioxide emissions, there are some limitations. The first is the availability and scope of data, which might affect the broad applicability of the study's results. Additionally, the current research may not have fully considered the indirect impacts of the digital economy's development on social behavior and lifestyles, which could also significantly influence carbon emissions. Future research directions could include expanding data sources and scope to provide a more comprehensive perspective; exploring in depth the specific impacts of digital technology on different economic sectors and lifestyles; examining the relationship between the development of the digital economy and other environmental indicators, such as air quality and water resource management; and assessing long-term impacts and trends. Through these studies, we can gain a more comprehensive understanding of the potential and challenges of the digital economy in promoting sustainable development.

## Data Availability

This study is based on empirical analysis using data sets from 107 resource-based cities in China from 2005 to 2021. The list of these cities is derived from the “National Plan for Sustainable Development of Resource-Based Cities (2013–2020)” (http://big5.www.gov.cn/gate/big5/www.gov.cn/zfwj/2013-12/03/content_2540070.htm). CO2 Emission Data: These data are sourced from various Chinese statistical yearbooks and related statistical materials, including the “China Energy Statistical Yearbook”, “China Industrial Statistical Yearbook”, “China Agricultural Statistical Yearbook”, “China Animal Husbandry Yearbook”, and “China Forestry and Grassland Statistical Yearbook”. These yearbooks can be accessed through the National Bureau of Statistics website (https://www.stats.gov.cn/). Digital Economy Data: These data are derived from the “China City Statistical Yearbook” and the “China Digital Inclusive Finance Index (2011–2021)” (https://idf.pku.edu.cn/), which is jointly compiled by the Digital Finance Research Center of Peking University and Ant Group. Other City Data: These are also sourced from the "China City Statistical Yearbook". For missing values, we used linear interpolation to complete the data. Since the data are mainly sourced from publicly accessible third-party resources, we cannot provide direct download links or datasets. During this study, we ensured that all data used can be accessed by other researchers through official channels, without the use of any special access privileges.

## References

[1] Qureshi S (2012). As the global digital divide narrows, who is being left behind?. Inf. Technol. Dev..

[2] Zhao, H. Global ICT development and ITU. In *2008 Third International Conference on Communications and Networking in China*, pp. vii-viii. 10.1109/chinacom.2008.4684950 (2008).

[3] Zhang J, Li J, Ye D, Sun C (2022). The impact of digital economy of resource-based city on carbon emissions trading by blockchain technology. Comput. Intell. Neurosci..

[4] Guo B, Feng W, Lin J (2024). Does market-based environmental regulation improve the residents’ health: Quasi-natural experiment based on DID. Inq. J. Health Care Organ. Provis. Financ..

[5] Hu F, Ma Q, Hu H, Zhou KH, Wei S (2024). A study of the spatial network structure of ethnic regions in Northwest China based on multiple factor flows in the context of COVID-19: Evidence from Ningxia. Heliyon.

[6] Li X, Liu J, Ni P (2021). The impact of the digital economy on CO2 emissions: A theoretical and empirical analysis. Sustainability.

[7] Liu L, Zhang Y, Gong X, Li M, Li X, Ren D, Jiang P (2022). Impact of digital economy development on carbon emission efficiency: A spatial econometric analysis based on Chinese provinces and cities. Int. J. Environ. Res. Public Health.

[8] Lyu K, Yang S, Zheng K, Zhang Y (2023). How does the digital economy affect carbon emission efficiency? Evidence from energy consumption and industrial value chain. Energies.

[9] Zha Q, Huang C, Kumari S (2022). The impact of digital economy development on carbon emissions—Based on the Yangtze River Delta urban agglomeration. Front. Environ. Sci..

[10] Obama B (2017). The irreversible momentum of clean energy. Science.

[11] Köne AÇ, Büke T (2010). Forecasting of CO2 emissions from fuel combustion using trend analysis. Renew. Sustain. Energy Rev..

[12] Guo B, Wang Y, Feng Y, Liang C, Tang L, Yao X, Hu F (2022). The effects of environmental tax reform on urban air pollution: A quasi-natural experiment based on the environmental protection tax law. Front. Public Health.

[13] Wei S, Yuwei W, Chongchong Z (2018). Forecasting CO2 emissions in Hebei, China, through moth-flame optimization based on the random forest and extreme learning machine. Environ. Sci. Pollut. Res..

[14] Sitch S, Friedlingstein P, Gruber N, Jones S, Murray-Tortarolo G, Ahlström A, Doney S, Graven H, Heinze C, Huntingford C, Levis S, Levy P, Lomas M, Poulter B, Viovy N, Zaehle S, Zeng N, Arneth A, Bonan G, Bopp L, Canadell J, Chevallier F, Ciais P, Ellis R, Gloor M, Peylin P, Piao S, Quéré C, Smith B, Zhu Z, Myneni R (2015). Recent trends and drivers of regional sources and sinks of carbon dioxide. Biogeosciences.

[15] Athanassiadis A, Christis M, Bouillard P, Vercalsteren A, Crawford R, Khan A (2018). Comparing a territorial-based and a consumption-based approach to assess the local and global environmental performance of cities. J. Clean. Prod..

[16] Zhang M, Tan F, Lu Z (2014). Resource-based cities (RBC): A road to sustainability. Int. J. Sustain. Dev. World Ecol..

[17] Tan J, Zhang P, Lo K, Li J, Liu S (2016). The urban transition performance of resource-based cities in Northeast China. Sustainability.

[18] Hou Y, Yin G, Chen Y (2022). Environmental regulation, financial pressure and industrial ecological efficiency of resource-based cities in China: Spatiotemporal characteristics and impact mechanism. Int. J. Environ. Res. Public Health.

[19] Shen X, Zhao H, Yu J, Wan Z, He T, Liu J (2022). Digital economy and ecological performance: Evidence from a spatial panel data in China. Front. Environ. Sci..

[20] Linkov I, Trump B, Poinsatte-Jones K, Florin M (2018). Governance strategies for a sustainable digital world. Sustainability.

[21] Creutzig F, Acemoglu D, Bai X, Edwards P, Hintz M, Kaack L, Kılkış Ş, Kunkel S, Luers A, Milojevic-Dupont N, Rejeski D, Renn J, Rolnick D, Rosol C, Russ D, Turnbull T, Verdolini E, Wagner F, Wilson C, Zekar A, Zumwald M (2022). Digitalization and the anthropocene. Annu. Rev. Environ. Resour..

[22] Herman E (2022). The interplay between digital entrepreneurship and sustainable development in the context of the EU digital economy: A multivariate analysis. Mathematics.

[23] Yu Z, Wan Y (2023). Can the growth of the digital economy be beneficial for urban decarbonization? A study from Chinese cities. Sustainability.

[24] Bai L, Guo T, Xu W, Liu Y, Kuang M, Jiang L (2023). Effects of digital economy on carbon emission intensity in Chinese cities: A life-cycle theory and the application of non-linear spatial panel smooth transition threshold model. Energy Policy.

[25] Zhang R, Liu H, Xie K, Xiao W, Bai C (2024). Toward a low carbon path: Do E-commerce reduce CO2 emissions? Evidence from China. J. Environ. Manag..

[26] Xu N, Zhang H, Li T, Ling X, Shen Q (2022). How big data affect urban low-carbon transformation—A quasi-natural experiment from China. Int. J. Environ. Res. Public Health.

[27] Wei M, Yin X (2024). Broadband infrastructure and urban carbon emissions: Quasi-experimental evidence from China. Urban Clim..

[28] Castro GDR, Fernandez MCG, Colsa AU (2021). Unleashing the convergence amid digitalization and sustainability towards pursuing the sustainable development goals (SDGs): A holistic review. J. Clean. Prod..

[29] Jing S, Wu F, Shi E, Wu X, Du M (2023). Does the digital economy promote the reduction of urban carbon emission intensity?. Int. J. Environ. Res. Public Health.

[30] Yu Z, Liu S, Zhu Z (2022). Has the digital economy reduced carbon emissions?: Analysis based on panel data of 278 cities in China. Int. J. Environ. Res. Public Health.

[31] Zhou B, Zhao H, Yu J, He T, Liu J (2022). Does the growth of the digital economy boost the efficiency of synergistic carbon-haze governance? Evidence from China. Front. Environ. Sci..

[32] Sun X, Chen Z, Shi T, Yang G, Yang X (2021). Influence of digital economy on industrial wastewater discharge: Evidence from 281 Chinese prefecture-level cities. J. Water Clim. Change.

[33] Wang X, Sun X, Zhang H, Ahmad M (2022). Digital economy and environmental quality: Insights from the spatial Durbin model. Int. J. Environ. Res. Public Health.

[34] Khaw-ngern K (2021). A digital circular economy for SDG 11 and SDG 12. PAE.

[35] Sun J, Wu X (2023). Research on the mechanism and countermeasures of digital economy development promoting carbon emission reduction in Jiangxi province. Environ. Res. Commun..

[36] Chen P (2022). Relationship between the digital economy, resource allocation and corporate carbon emission intensity: New evidence from listed Chinese companies. Environ. Res. Commun..

[37] Shindell D, Kuylenstierna JC, Vignati E, van Dingenen R, Amann M, Klimont Z, Fowler D (2012). Simultaneously mitigating near-term climate change and improving human health and food security. Science.

[38] Töbelmann D, Wendler T (2020). The impact of environmental innovation on carbon dioxide emissions. J. Clean. Prod..

[39] Weber CL, Matthews HS (2008). Quantifying the global and distributional aspects of American household carbon footprint. Ecol. Econ..

[40] Ghazali Z, Zahid M, Kee TS, Ibrahim MY (2016). A step towards sustainable society: The awareness of carbon dioxide emissions, climate change and carbon capture in Malaysia. Int. J. Econ. Financial Issues.

[41] Chen X, Huang B, Lin CT (2019). Environmental awareness and environmental Kuznets curve. Econ. Model..

[42] Anwar A, Younis M, Ullah I (2020). Impact of urbanization and economic growth on CO2 emission: A case of far east Asian countries. Int. J. Environ. Res. Public Health.

[43] Duarte R, Feng K, Hubacek K, Sánchez-Chóliz J, Sarasa C, Sun L (2016). Modeling the carbon consequences of pro-environmental consumer behavior. Appl. Energy.

[44] Wang Y, Sun M, Yang X, Yuan X (2016). Public awareness and willingness to pay for tackling smog pollution in China: A case study. J. Clean. Prod..

[45] Perera F (2018). Pollution from fossil-fuel combustion is the leading environmental threat to global pediatric health and equity: Solutions exist. Int. J. Environ. Res. Public Health.

[46] Dyer R, Spanellis A, Harviainen JT (2021). Gamified emissions through the wisdom of crowds. Transforming Society and Organizations through Gamification: From the Sustainable Development Goals to Inclusive Workplaces.

[47] Tan J, Chen L (2022). Spatial effect of digital economy on particulate matter 2.5 in the process of smart cities: Evidence from prefecture-level cities in China. Int. J. Environ. Res. Public Health.

[48] Cong JH, Liu XM, Zhao XR (2014). Boundary delineation and measurement methods of urban carbon emissions accounting. China Popul. Resour. Environ..

[49] Zhao T, Zhang Z, Liang SK (2020). Digital economy, entrepreneurship activity and high-quality development: Empirical evidence from Chinese cities. Manag. World.

[50] Jiang T (2022). Mediating and moderating effects in empirical research on causal inference. China Ind. Econ..

[51] Hu J, Yu X, Han Y (2023). Can ESG ratings promote corporate green transformation? Evidence from a multi-time point difference-in-differences approach. Quant. Econ. Tech. Econ. Res..

[52] Li X, Dang L, Zhao CY (2022). Digital transformation, integration into global innovation networks and innovation performance. China Ind. Econ..

[53] Lewbel A (1997). Constructing instruments for regressions with measurement error when no additional data are available, with an application to patents and R&D. Econom. J. Econom. Soc..

[54] Wu L, Yang M, Sun K (2022). The impact of public environmental awareness on corporate and government environmental governance. China Popul. Resour. Environ..

[55] Feng Y, Guo B, Wang X, Hu F (2024). Facilitating or inhibiting? The impact of environmental information disclosure on enterprise investment value. Environ. Sci. Pollut. Res..

[56] Chen SY, Chen DK (2018). Haze pollution, government governance and high-quality economic development. Econ. Res. J..

[57] Song X, Tian Z, Ding C, Liu C, Wang W, Zhao R, Xing Y (2022). Digital economy, environmental regulation, and ecological well-being performance: A provincial panel data analysis from China. Int. J. Environ. Res. Public Health.

[58] Li Y, Yang X, Ran Q, Wu H, Irfan M, Ahmad M (2021). Energy structure, digital economy, and carbon emissions: evidence from China. Environ. Sci. Pollut. Res. Int..

[59] Chen X, Mao S, Lv S, Fang Z (2022). A study on the non-linear impact of digital technology innovation on carbon emissions in the transportation industry. Int. J. Environ. Res. Public Health.

[60] Ding Y, Yang Y (2023). The influence of digital development on China’s carbon emission efficiency: In the view of economic and environmental balance. Front. Environ. Sci..

[61] Liao Z, Ru S, Cheng Y (2023). A simulation study on the impact of the digital economy on CO2 emission based on the system dynamics model. Sustainability.

[62] Yan J, Lu Q, Tang J, Chen L, Hong J, Broyd T (2022). Digital tools for revealing and reducing carbon footprint in infrastructure, building, and city scopes. Buildings.

[63] Batmunkh A (2022). Carbon footprint of the most popular social media platforms. Sustainability.

[64] Zhong R, He Q, Qi Y (2022). Digital economy, agricultural technological progress, and agricultural carbon intensity: Evidence from China. Int. J. Environ. Res. Public Health.

[65] Pedram M (2009). Green computing: Reducing energy cost and carbon footprint of information processing systems. Proc. ACM Int. Conf. Comput. Front..

[66] Guo B, Feng Y, Lin J, Wang X (2024). New energy demonstration city and urban pollutant emissions: An analysis based on a spatial difference-in-differences model. Int. Rev. Econ. Finance.

[67] Chang X, Li J (2022). Effects of the digital economy on carbon emissions in China: A spatial Durbin econometric analysis. Sustainability.

[68] Wang H, Wei W (2020). Coordinating technological progress and environmental regulation in CO2 mitigation: The optimal levels for OECD countries & emerging economies. Energy Econ..

